# Iptacopan for Immune Thrombocytopenia and Cold Agglutinin Disease: A Global Phase 2 Basket Clinical Trial

**DOI:** 10.1002/ajh.70147

**Published:** 2025-12-09

**Authors:** Alexander Röth, Wilma Barcellini, Christine Ademokun, Junho Jang, Maria Luisa Lozano, David Valcarcel Ferreiras, Cristina Pascual‐Izquierdo, Shripad Chitnis, Sofiya Matviykiv, Alessandra Vitaliti, Chi Chen, Vasiliki Katsanou, Raghav Chawla, Hanny Al‐Samkari

**Affiliations:** ^1^ Department of Hematology and Stem Cell Transplantation, West German Cancer Center, University Hospital Essen University Duisburg‐Essen Essen Germany; ^2^ Department of Hematology Fondazione IRCCS Ca' Granda Ospedale Maggiore Policlinico Milan Italy; ^3^ Hammersmith Hospital Imperial College Healthcare NHS Trust London UK; ^4^ Samsung Medical Center Seoul Republic of Korea; ^5^ Servicio de Hematología, Hospital Universitario Morales Meseguer, Instituto Murciano de Investigación Sanitaria (IMIB)‐Pascual Parrilla Universidad de Murcia Murcia Spain; ^6^ Hematology Department, Vall Hebron Institute of Oncology (VHIO) Vall d'Hebron University Hospital Barcelona Spain; ^7^ Servicio de Hematología Hospital General Universitario Gregorio Marañon, Instituto de Investigación Gregorio Marañon Madrid Spain; ^8^ Novartis Biomedical Research Cambridge Massachusetts USA; ^9^ Novartis Biomedical Research Basel Switzerland; ^10^ China Novartis Institutes for Biomedical Research Co Ltd Shanghai China; ^11^ Division of Hematology, Massachusetts General Hospital Harvard Medical School Boston Massachusetts USA

**Keywords:** alternative complement pathway, cold agglutinin disease, complement inhibition, hemolytic anemia, immune thrombocytopenia, Iptacopan

## Abstract

Iptacopan is a first‐in‐class, oral, selective inhibitor of complement factor B that has demonstrated positive efficacy across several complement‐driven diseases. Here we evaluate the efficacy and safety of iptacopan monotherapy in adult patients with primary immune thrombocytopenia (ITP) and primary cold agglutinin disease (CAD). We performed a global, multicenter, phase 2 basket study enrolling patients with primary ITP or CAD after failure of ≥ 1 unique prior therapies. Primary endpoints were platelet response (≥ 50 × 10^9^/L) for ITP and hemoglobin response (≥ 1.5 g/dL increase) for CAD sustained for ≥ 2 consecutive weeks during the first 12 weeks of iptacopan treatment, without the use of rescue therapy. Other endpoints included time to first response, duration of response, pharmacokinetics, safety/tolerability, and FACIT‐Fatigue. Nineteen patients were treated with iptacopan (9 ITP, 10 CAD). Among patients with CAD, most showed improvements in hemoglobin levels, with a mean increase of 2.2 g/dL from baseline to week 12; five (50%) patients achieved the primary endpoint. Improvements were also observed for other outcomes in CAD, including lactate dehydrogenase, bilirubin, reticulocytes, and FACIT‐Fatigue. Conversely, no patients with ITP met the primary endpoint. Most treatment‐emergent adverse events (TEAEs) were mild, the most common being headache (21%), asthenia (16%), fatigue (16%), and petechiae (16%). Iptacopan monotherapy demonstrated encouraging preliminary efficacy in primary CAD, while no protocol‐defined responses were observed in primary ITP. Iptacopan may represent a promising oral option for CAD and was well tolerated in both ITP and CAD with no unexpected safety signals.

**Trial Registration:** ClinicalTrials.gov identifier: NCT05086744

## 1 Introduction

Immune thrombocytopenia (ITP) and cold agglutinin disease (CAD) are autoimmune hematologic disorders causing thrombocytopenia and hemolytic anemia, respectively [1]. ITP results in IgG platelet autoantibody‐mediated platelet destruction that leads to bleeding and constitutional symptoms. ITP has an annual incidence of 1.9–3.9 per 100 000 person‐years, with higher rates in women and in older age groups [2]. The initial treatment options for ITP, beyond watchful waiting and supportive therapy, include corticosteroids, intravenous immunoglobulin (IVIg), and anti‐D immunoglobulin (for Rh‐positive patients), with subsequent treatment options including thrombopoietin receptor agonists (TPO‐RAs), rituximab, fostamatinib and splenectomy [3]. While platelet destruction is thought to occur primarily through antibody‐dependent cellular cytotoxicity in the spleen, evidence suggests that complement activation plays a significant role in platelet destruction in at least a subset of ITP patients, likely through complement fixation by platelet autoantibodies and activation of the classical complement pathway [4, 5, 6]. Consistent with this, several inhibitors of the classical complement pathway, including sutimlimab, have shown preliminary efficacy in patients with refractory primary ITP [7, 8].

Primary CAD is characterized by hemolytic anemia triggered by cold temperatures. It is a rare disease, with an annual incidence of one case per million and a prevalence of 16 per million [9, 10]. CAD predominantly affects adults, with a median age of 67 at presentation. Cold‐reacting autoantibodies, primarily of the IgM type, target red blood cell (RBC) surface antigens, leading to hemolysis via activation of the classical complement pathway. Several complement inhibitors have shown efficacy in treating refractory primary CAD [11, 12, 13, 14, 15], with the classical complement pathway inhibitor sutimlimab being the first and only approved treatment [16, 17, 18]. Standard‐of‐care options for CAD, beyond watchful waiting and supportive therapy (including RBC transfusions) [19], include either sutimlimab or early use of rituximab, with or without bendamustine.

Despite available treatment options for both primary ITP and CAD, there remains an unmet need for effective therapies, including therapies capable of achieving lasting remissions in relapsed or refractory patients. Moreover, there are no oral therapy options available for CAD.

Iptacopan (LNP023; Novartis Pharma AG, Switzerland) is a novel small‐molecule selective inhibitor of complement factor B, which is a crucial component of the complement alternative pathway. By inhibiting both the alternative pathway C3 and C5 convertases, iptacopan blocks C3 activation and the formation of the membrane attack complex (MAC) [20]. Moreover, iptacopan inhibits the amplification loop of the complement system, which is a significant contributor to the overall MAC formation in vitro, regardless of the initiating trigger of complement activation [21]. By targeting this amplification mechanism, iptacopan has the potential to provide therapeutic benefit even in diseases where complement activation occurs primarily via the classical pathway [22]. Iptacopan has shown positive efficacy and has been approved by the US Food and Drug Administration (FDA) across several complement‐driven diseases, including paroxysmal nocturnal hemoglobinuria (PNH) [23, 24], primary immunoglobulin A nephropathy (IgAN) [25, 26], and C3 glomerulopathy (C3G) [27, 28]. Importantly, iptacopan offers the convenience of oral administration.

The objective of this phase 2 proof‐of‐concept study was to investigate iptacopan monotherapy in treating autoimmune hematological disorders associated with complement activation. The study was designed as a basket study, focusing on two indications, primary ITP and primary CAD.

## 2 Materials and Methods

### 2.1 Study Design

**FIGURE 1:**
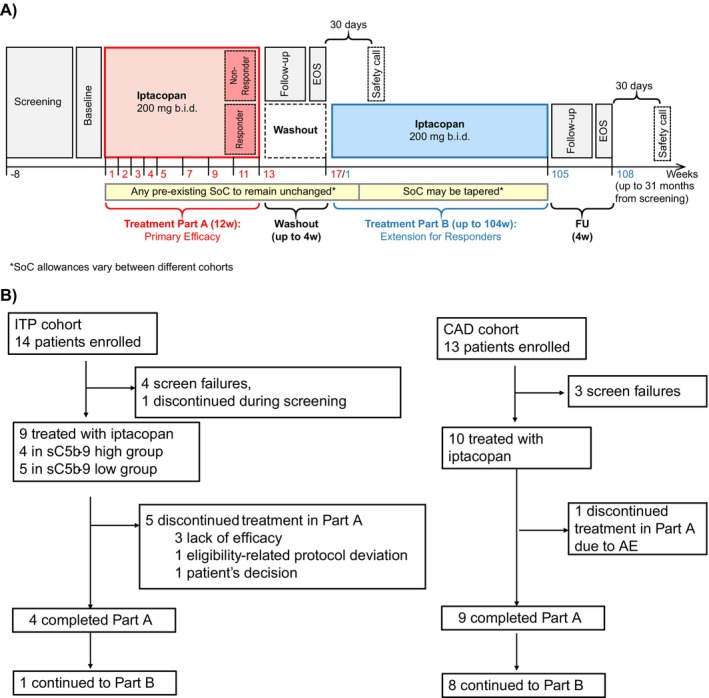
(A) Study design. (B) Patient disposition. AE, adverse event; b.i.d, twice a day; CAD, cold agglutinin disease; EOS, end of study; FU, follow‐up; ITP, immune thrombocytopenia; SoC, standard of care; 4w, 4 weeks; 12w, 12 weeks.

This was a multicenter, open‐label, non‐randomized, phase 2 basket study (NCT05086744) to assess the efficacy, safety and pharmacokinetics of iptacopan monotherapy in adult patients with primary ITP and primary CAD. The study was conducted in 8 centers across 6 countries (Germany, Italy, South Korea, Spain, United Kingdom, and USA) and included two cohorts: primary ITP and primary CAD. Patients were enrolled between December 2021 and June 2023. Patients with primary ITP were stratified into two subgroups (with a cap of approximately 10 patients each) based on sC5b‐9 levels at screening, using 200 ng/mL as a cutoff for high versus low complement activation. This threshold was selected by the sponsor based on internal and external analyses of ITP patient samples [5]. The study consisted of a screening period, a 12‐week treatment period (Part A), a washout period (for responders; ≤ 4 weeks) or follow‐up period (for non‐responders) after Part A, and an optional treatment extension period for up to 24 months (Part B) for responders as well as for patients who had a clinically meaningful response as assessed by the investigator despite not meeting the primary endpoint. Per the protocol, the washout period could be shortened if participants' blood counts fell below certain thresholds (platelets < 30 × 10^9^/L for ITP and Hb < 10 g/dL for CAD). The total study duration from screening until end of study (EOS) was approximately 6 months for patients discontinuing after Part A and up to 31 months for patients proceeding with Part B (Figure 1A). In both cohorts, patients received iptacopan 200 mg orally twice daily (b.i.d.) during the treatment periods.

### 2.2 Patients

For the ITP cohort, eligibility criteria included adult patients with a diagnosis of persistent or chronic primary ITP (diagnosed at least 3 months prior to baseline), with evidence of sustained thrombocytopenia (platelets < 30 × 10^9^/L), as documented at baseline plus in ≥ 2 additional assessments separated by at least 1 week in the prior 3 months, after at least one unique prior ITP‐directed therapy. Transfusions of blood products were not counted as prior therapies. Patients with secondary ITP, as may arise in the setting of certain rheumatologic disorders, immunodeficiency syndromes, infections, malignancies, and drug treatments were excluded. No concomitant ITP‐directed background therapy was permitted, with the exception of either a single TPO‐RA or low‐dose corticosteroid (prednisone‐equivalent of ≤ 10 mg daily) with a stable dosage for at least 4 weeks prior to baseline. Patients with abnormal coagulation screening labs (prothrombin time or activated partial thromboplastin time) were excluded.

For the CAD cohort, eligibility criteria included adult patients with a diagnosis of primary CAD, including CAD arising in the setting of a low‐grade lymphoproliferative disorder, with a positive direct antiglobulin test for C3d only (or predominantly), a cold agglutinin titer of ≥ 64 at 4°C, and evidence of sustained anemia (Hb < 10 g/dL) as documented at baseline plus in ≥ 2 additional assessments separated by at least 1 week in the prior 3 months, and of ongoing hemolysis after at least one unique prior CAD‐directed therapy. Patients with secondary CAD, as may arise in the setting of certain infections, autoimmune disorders, and malignancies (with the exception of a low‐grade lymphoproliferative disorder) were excluded and no concomitant CAD‐directed background therapy was permitted.

For all patients, vaccination against *Neisseria meningitidis* , *Streptococcus pneumoniae* and, if available, against *Haemophilus influenzae* infections was required according to local regulations prior to the start of treatment.

### 2.3 Study Objectives

The primary objective of the study was to assess the ability of iptacopan to induce a clinically meaningful increase in platelet count (ITP) or hemoglobin levels (CAD). For ITP, the primary endpoint of a clinically meaningful response was defined as a platelet count of ≥ 50 × 10^9^/L sustained for at least two consecutive weeks during the main, 12‐week treatment part (Part A) without the use of rescue therapy. For CAD, the primary endpoint of a clinically meaningful response was defined as an Hb increase of ≥ 1.5 g/dL above baseline that was sustained for at least two consecutive weeks during Part A without the use of rescue therapy. Patients were considered responders if they met the primary endpoint. The administration of rescue therapy or discontinuation before meeting the primary endpoint criteria classified the patient as a non‐responder.

Secondary objectives included the assessments of time to first response, duration and magnitude of response, relevant disease biomarkers (for CAD cohort; lactate dehydrogenase [LDH], bilirubin, reticulocytes, and haptoglobin), pharmacokinetics (PK) of iptacopan, and safety/tolerability. Plasma samples for analysis of PK parameters were obtained and evaluated in all participants receiving iptacopan. Disease biomarkers including LDH and total bilirubin were quantified in serum by enzymatic and diazo‐based colorimetric methods, respectively, while haptoglobin was measured by immunoturbidimetry. Reticulocytes were counted in whole blood using an automated system that stains cellular RNA and classifies cells based on laser scatter and absorption characteristics. Detailed descriptions of disease biomarker assays are available in the Supplemental Appendix. Pharmacokinetic sampling for iptacopan concentration measurements was conducted on days 15 and 57 from 0 to 6 h post dose.

Exploratory objectives included the assessments of quality of life using the Functional Assessment of Chronic Illness Therapy (FACIT)‐Fatigue scale [29], additional disease biomarkers for CAD (including C3d deposition on RBC, measured by flow cytometry), and complement pathway biomarkers (e.g., Factor Bb, Wieslab, sC5b‐9, C3, C4 and C4d) to evaluate the impact of iptacopan on the classical and alternative pathways. C3 and soluble (s)C5b‐9 are general biomarkers of complement activation, with C3 acting as a central component consumed during pathway activation and sC5b‐9 reflecting terminal pathway activity, regardless of which complement pathway is triggered. Conversely, C4 is specific to the classical and lectin pathways, and its cleavage fragment, C4d, indicates activation of either of these. Finally, Factor Bb and the Wieslab alternative pathway are specific biomarkers for the complement alternative pathway. Factor Bb, sC5b‐9, Wieslab alternative pathway, and C4d were measured using sandwich ELISA techniques with specific monoclonal antibodies and colorimetric or fluorescent detection. C3 and C4 concentrations were determined by immunoturbidimetric assays on the Abbott Architect system, wherein turbidity from antibody–antigen complexes was measured. Detailed descriptions of complement pathway biomarker assays are available in the Supplemental Appendix.

For patients moving on to Part B, the long‐term effects on key hematological parameters were also assessed as an exploratory objective.

### 2.4 Statistical and Pharmacokinetic Analyses

Data analysis was conducted separately for each cohort (ITP and CAD), with a stratification for the ITP cohort based on sC5b‐9 levels at screening as described above.

The full analysis set (FAS) and safety set included all patients who received at least one dose of study treatment. The pharmacodynamic (PD) analysis set included all patients with available PD data who received any study drug and had no protocol deviations with relevant impact on PD data. The PK analysis set included all patients with at least one available valid PK concentration measurement who received any study drug and had no protocol deviations with relevant impact on PK data.

Statistical evaluation of primary and secondary efficacy data, PK and PD endpoints was descriptive, with no hypothesis testing performed. The secondary efficacy endpoints were summarized in responders only. Safety data analysis included all patients, focusing on AEs, laboratory values, ECGs, and vital signs.

For the ITP cohort, positive efficacy (or proof‐of‐concept) was defined as an observed response rate (ORR) of ≥ 30% in all patients, or of ≥ 50% in patients with high activated complement at screening (i.e., sC5b‐9 high). For the CAD cohort, positive efficacy was defined as an ORR of ≥ 50%. Approximately 20 ITP patients (10 in each subgroup) and 10 CAD patients were planned to be enrolled in this study. For ITP, with a total of 20 planned patients, the study had an 80% probability of meeting the target ORR of ≥ 30% at a true ORR of 37%. For CAD and for the complement‐activated ITP subgroup, with a total of 10 planned patients each, the study had an 80% probability of meeting the target ORR of ≥ 50% at a true ORR of 58%.

Iptacopan concentration was determined by a validated LC–MS/MS method with a previously established lower limit of quantification of 1 ng/mL. Due to the limited sampling interval of 6 h, the calculated PK parameters were determined using the actual recorded sampling times and non‐compartmental method(s) with Phoenix WinNonlin (version 8 or higher) and were limited to the following: C_max_, T_max_, AUC_last_ and AUC_tau_. AUC_tau_ was calculated by the “last observation carried forward” approach, assuming that, at steady state, the iptacopan plasma concentration at T = 12 h (tau) was the same as the profile's corresponding pre‐dose (T = 0) value. This approach was applied since sampling times were limited to 6 h post dose and the extrapolation of AUC_last_ (0–6 h) to AUC_tau_ (0–12 h) was likely to exceed the accepted value of 20% of the total AUC and be based on an unreliable half‐life estimate. The linear trapezoidal rule was used for AUC calculations.

Exploratory variables analyzed the effect of iptacopan on the complement pathway and disease biomarkers. The FAS was used for these analyses. Complement pathway biomarkers were analyzed in Part A only for both ITP and CAD (see Supplemental Appendix for assay details). Additional disease biomarkers were analyzed in Part A and Part B for CAD (see Supplemental Appendix for details). The FACIT‐Fatigue scale was used to assess patients' quality of life in both the cohorts [26].

## 3 Results

### 3.1 Demographic and Other Baseline Characteristics

Demographic and other baseline characteristics are summarized in Table 1. In the ITP cohort, 55.6% of patients were female, the median age was 42.0 (range: 20–78) years, and the median baseline platelet count was 15.0 × 10^9^/L (range: 5–38). Patients had received a median of 3.0 (range: 2–6) unique prior therapies, most commonly glucocorticoids, TPO‐RAs (romiplostim, eltrombopag), and rituximab. At the start of the study, 44.4% of patients with ITP were receiving background therapies as permitted by the protocol, including low‐dose glucocorticoids or romiplostim. All nine patients enrolled in the ITP cohort had received meningococcal and pneumococcal vaccinations before the start of iptacopan treatment as per the protocol requirements, and seven out of nine patients (77.8%) had also received vaccination against *Hemophilus influenzae* B. Further details regarding these vaccinations are shown in Table S1.

**TABLE 1:** Demographic and other baseline characteristics (Part A).

	ITP, sC5b‐9 high *N* = 4^a^	ITP, sC5b‐9 low *N* = 5	ITP, all *N* = 9	CAD *N* = 10	All patients *N* = 19
Age (years), median (range)	43.0 (25–78)	42.0 (20–70)	42.0 (20–78)	67.5 (52–82)	60.0 (20–82)
Sex, *n* (%)					
Female	1 (25.0)	4 (80.0)	5 (55.6)	10 (100)	15 (78.9)
Male	3 (75.0)	1 (20.0)	4 (44.4)	0	4 (21.1)
Race, *n* (%)					
Asian	2 (50.0)	1 (20.0)	3 (33.3)	0	3 (15.8)
White	2 (50.0)	4 (80.0)	6 (66.7)	10 (100)	16 (84.2)
Number of unique prior therapies, Mean (SD)	3.3 (1.89)	4.4 (1.82)	3.9 (1.83)	2.7 (1.64)	3.3 (1.79)
Background therapies, *n*	1 [prednisone]	3 [romiplostim]	4 [romiplostim, prednisone]	N/A	4
Baseline Hb in g/dL, median (range)	N/A	N/A	N/A	8.8 (6.8–9.9)	N/A
Baseline LDH in U/L, median (range)	N/A	N/A	N/A	434.0 (279–598)	N/A
Baseline bilirubin in μmol/L, median (range)	N/A	N/A	N/A	48.1 (20–141)	N/A
Baseline platelets (×10^9^/L), median (range)	16.5 (10–20)	5.0 (5–38)	15.0 (5–38)	N/A	N/A
Baseline sC5b‐9^b^ in ng/mL, median (range)	226.0 (207–313)	150 (150–174)	174.3 (150–313)	N/A	N/A
FACIT‐Fatigue^c^					
Mean (SD)	29.3 (15.82)	37.4 (11.46)	34.4 (12.81)	34.3 (8.96)	34.3 (10.49)
Median (range)	33.0 (12–43)	39.0 (22–52)	36.0 (12–52)	35.0 (16–44)	35.5 (12–52)

Abbreviations: CAD, cold agglutinin disease; ITP, immune thrombocytopenia; LDH, lactate dehydrogenase; SD, standard deviation.

^a^ Patient with sC5b‐9 high levels at screening was excluded from the PD analysis set due to an eligibility‐related protocol deviation (treated concomitantly with a higher than allowed dose of corticosteroids).

^b^ Baseline sC5b‐9 reflects levels determined at screening for stratification of ITP patients (ICON).

^c^ Scores on the FACIT‐Fatigue Scale range from 0 to 52, with higher scores indicating less fatigue.

In the CAD cohort, all patients were females, with a median age of 67.5 (range: 52–82) years, a median baseline Hb level of 8.8 g/dL (range: 6.8–9.9), and a median baseline LDH of 434.0 U/L (range: 279–598). Patients had received a median of 2.0 (range: 1–6) unique prior therapies, most commonly rituximab, glucocorticoids, alkylating agents (cyclophosphamide, bendamustine), and bortezomib. Five patients had received RBC transfusions within 12 months before starting iptacopan, with a median of 0.5 transfusions (range: 0–27) across all patients, and two patients had received RBC transfusions within 6 months before starting iptacopan (see Table S2 for further details regarding transfusion history). All 10 patients enrolled in the CAD cohort had received meningococcal and pneumococcal vaccinations before the start of iptacopan treatment as per the protocol requirements, and eight out of 10 patients (80.0%) had also received vaccination against *Hemophilus influenzae* B. Further details regarding these vaccinations are shown in Table S1.

Demographic and other baseline characteristics for Part B are summarized in Table S3.

### 3.2 Patient Disposition

Patient disposition for both cohorts is illustrated in Figure 1B. Fourteen patients were enrolled in the ITP cohort, with 9 patients treated (four in the sC5b‐9 high group and five in the sC5b‐9 low group), four screen failures, and one patient who discontinued during screening (patient's decision). Four patients completed the Part A treatment period, whereas five patients discontinued treatment: three due to lack of efficacy and one each due to an eligibility‐related protocol deviation and patient's decision. One patient continued to treatment extension in Part B (Figure 1B, Table S4). Among the nine treated ITP patients, the patient with the eligibility‐related protocol deviation was excluded from the PD analysis set. The median exposure to iptacopan was 64 days (range 22–85 days) in Part A, and the patient who moved on to Part B was treated for an additional 207 days.

Thirteen patients were enrolled in the CAD cohort, with 10 patients treated and three screen failures. Nine patients completed the Part A treatment period, whereas one patient discontinued treatment due to an AE (patient experienced a recurrence of breast cancer, which was considered unrelated to study treatment). Eight patients continued to Part B (Figure 1B, Table S4). All treated patients in Part A (*n* = 10) and Part B (*n* = 8) were included in all analysis sets. The median exposure to iptacopan was 84.5 days (range 16–92 days) in Part A and 345 days (range 53–631 days) in Part B.

### 3.3 Efficacy

#### 3.3.1 ITP Cohort

**FIGURE 2:**
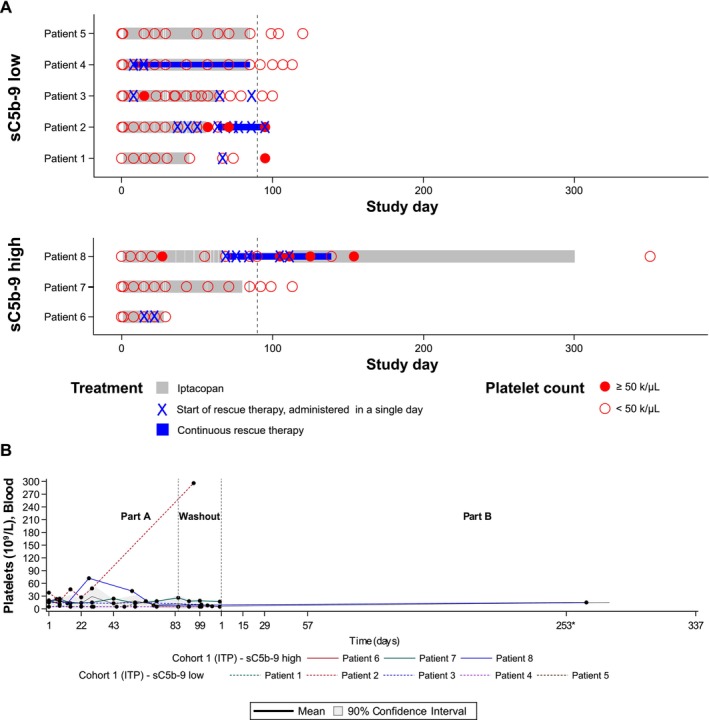
Swimmer plot and platelet count over time for ITP patients. (A) Swimmer plot for all ITP patients—Part A and Part B. (B) Overlaying individual line and arithmetic mean plots of platelet count over time ITP—Part A. Actual study days are shown for individual patients, whereas planned study visit days were considered for the calculation of mean and confidence interval. Visits occurring outside of the protocol‐defined visit schedule and early end‐of‐treatment visits have been matched to and are displayed as their closest planned visit as per the time window defined in the SAP. The confidence intervals for the raw value of biomarkers (LLN/LLOQ of which is non‐negative) are adjusted such that the negative lower limit is truncated to 0. ITP, immune thrombocytopenia; LLN/LLOQ, lower limit of normal/lower limit of quantification; SAP, statistical analysis plan.

As illustrated in Figure 2, among eight evaluable ITP patients, none met the primary endpoint of a platelet count increase to ≥ 50 × 10^9^/L sustained for at least two consecutive weeks without the use of rescue therapy. Five (62.5%) patients required rescue therapy while being treated with iptacopan in Part A, whereas two patients completed the full Part A treatment period without the need for rescue therapy but did not meet the criteria to be considered responders. The mean platelet count did not show a significant improvement in the absence of rescue therapy, and only one patient (patient 8; sC5b‐9 high) crossed the response threshold of platelet count of > 50 × 10^9^/L on a single occasion. Although this patient did not meet the protocol's definition of a responder, he continued treatment with iptacopan in Part B as he derived clinical benefit as per the investigator's assessment. With the exception of this patient, no discernible differences were observed between the sC5b‐9 high versus low subgroups. Ultimately, patient enrollment in the ITP cohort was halted early based on preliminary results suggesting a lack of efficacy in this patient population.

#### 3.3.2 CAD Cohort

**FIGURE 3:**
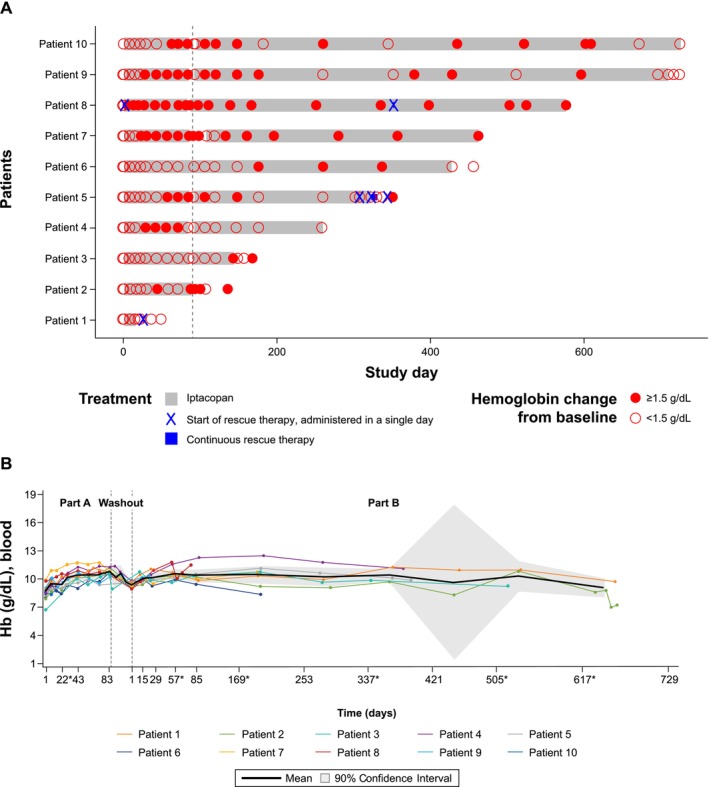
Swimmer plot and hemoglobin levels over time for CAD patients. (A) Swimmer plot for all patients CAD—Part A and Part B. (B) Overlaying individual line and arithmetic mean plots of hemoglobin over time CAD—Part A and Part B. Actual study days are shown for individual patients, whereas planned study visit days were considered for the calculation of mean and confidence interval. *Visits occurring outside of the protocol‐defined visit schedule and early end‐of‐treatment visits have been matched to and are displayed as their closest planned visit as per the time window defined in the SAP. The confidence intervals for the raw value of biomarkers (LLN/LLOQ of which is non‐negative) are adjusted such that the negative lower limit is truncated to 0. CAD, cold agglutinin disease; LLN/LLOQ, lower limit of normal/lower limit of quantification; SAP, statistical analysis plan.

As illustrated in Figure 3, among 10 evaluable CAD patients, 5 met the primary endpoint of a Hb level increase of ≥ 1.5 g/dL above baseline sustained for at least two consecutive weeks without the use of rescue therapy. A total of eight patients (80%) reached Hb levels of ≥ 10 g/dL by the end of Part A (Week 12), with six (60%) achieving an increase of ≥ 1.5 g/dL (including patient #8 who did not meet the primary endpoint; see below) and four (40%) achieving an increase of ≥ 2 g/dL above baseline. The mean Hb level showed an upward trend over time as well, with a mean increase from baseline of 2.2 g/dL at week 12 (Table 2). The median time to first response (Hb ≥ 1.5 g/dL above baseline) for the five responders was 29.0 days (range: 23–63 days), and the median duration of response in Part A was 56.0 days (range 28–63 days). Of note, patient #8 received an RBC transfusion on day 2 of the Part A treatment period and was therefore considered a non‐responder. However, the Hb levels of that patient showed a sustained increase of ≥ 1.5 g/dL from baseline during the entire Part A treatment period.

**TABLE 2:** Hemolytic biomarkers at week 12 in CAD patients.

Hemolytic biomarker	Baseline mean (range)	Week 12, mean (range)	Change from baseline to Week 12, mean (range)
Hb, g/dL (*n* = 8)	8.6 (6.8–9.9)	10.8 (10.4–11.3)	+2.2 (0.6–3.8)
LDH, U/L (*n* = 6)	489.5 (332–598)	211.7 (143.0–344.0)	−277.8 (−409.0 to −157.0)
Bilirubin, μmol/L (*n* = 8)	64.4 (30–141)	48.8 (24.2–115.0)	−15.6 (−33.4 to 5.9)
Haptoglobin, g/L (*n* = 8)	0.03 (0–0)	0.2 (0.0–0.8)	+0.1 (0.0–0.7)
Reticulocyte count, 10^9^/L (*n* = 8)	184.2 (124–272)	146.8 (97.7–222.0)	−37.5 (−57.0 to −8.0)

Abbreviations: CAD, cold agglutinin disease; Hb, hemoglobin; LDH, lactate dehydrogenase.

**FIGURE 4:**
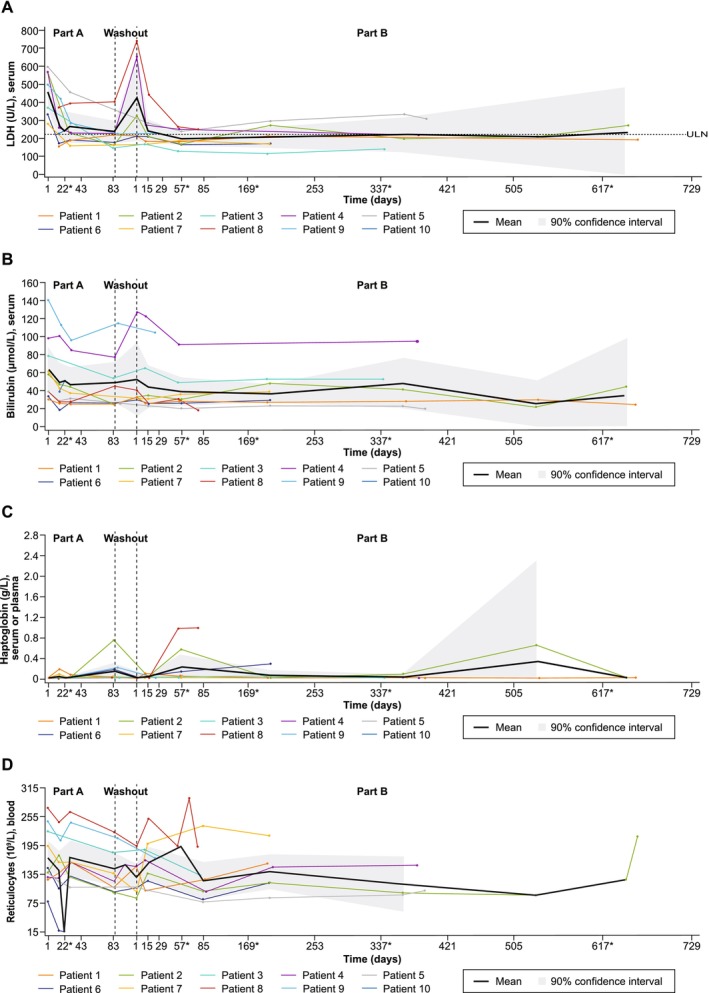
Key hemolytic markers over time in CAD patients. (A) serum LDH (B) serum bilirubin (C) haptoglobin (D) reticulocytes. Actual study days are shown for individual patients, whereas planned study visit days were considered for the calculation of mean and confidence interval. *Visits occurring outside of the protocol‐defined visit schedule and early end‐of‐treatment visits have been matched to and are displayed as their closest planned visit as per the time window defined in the SAP. The confidence intervals for the raw value of biomarkers (LLN/LLOQ of which is non‐negative) are adjusted such that the negative lower limit is truncated to 0. CAD, cold agglutinin disease; LDH, lactate dehydrogenase; LLN/LLOQ, lower limit of normal/lower limit of quantification; SAP, statistical analysis plan.

Treatment with iptacopan also resulted in an improvement in hemolytic parameters. In particular, all patients experienced a decrease in LDH from baseline to the end of Part A (week 12) (Figure 4A), and improvements were also observed in Part A for other hemolytic biomarkers, including bilirubin, haptoglobin and reticulocyte counts (Table 2; Figure 4B–D).

There was no significant effect on exploratory disease biomarkers like C3d deposition on RBC, and no changes were observed in the direct Coombs test (DAT) and cold agglutinin titers (data not shown).

Eight patients continued to Part B, including all five patients who met the primary endpoint and three who had an investigator‐assessed clinical benefit. The mean duration of washout between Part A and Part B was 8.4 days (range:0–23). The Hb levels dropped rapidly as a result of treatment pause (washout) at the end of Part A. Upon resumption of iptacopan treatment in Part B, Hb levels re‐increased rapidly to similar levels as in Part A and the increase was sustained in most patients throughout the Part B treatment period (Figure 3A,B). Likewise, the improvements in hemolytic parameters were maintained in most patients in Part B (Figure 4). The median (range) duration of iptacopan exposure in Part B was 345.0 (53.0–631.0) days. Two patients received rescue therapy in Part B. One of the patients (patient #8) received an RBC transfusion during the treatment period but continued with iptacopan treatment. The other patient (patient #5) received an RBC transfusion, discontinued iptacopan treatment due to lack of efficacy, and started sutimlimab during the follow‐up period. The other reasons for discontinuation in Part B included an AE (patient #3) and the early study termination for the remaining six patients. Indeed, following the evaluation of the primary and secondary endpoints, the study was terminated earlier than planned; this early termination was due to a business decision and not a consequence of any safety concern.

### 3.4 Pharmacokinetics and Pharmacodynamics

Iptacopan was rapidly absorbed with a median (range) T_max_ of 1 (1–2) hours and 1.97 (1–2) hours post‐dose on PK sampling days Day 15 and Day 57 respectively. Exposure to iptacopan (C_max_ and AUC) at steady state was broadly comparable on both PK sampling days (Day 15 and Day 57) and between both cohorts (ITP, CAD) with low to moderate inter‐patient variability (%CV 15%–35%). Pre‐dose iptacopan concentrations (i.e., PK trough, total concentration) were above the established EC90 (~900 ng/mL) based on the Wieslab assay, suggesting near full target (i.e., Factor B) inhibition throughout the dosing interval. Iptacopan plasma concentrations at different timepoints are shown in Table S5, and individual and arithmetic mean plasma concentration‐time profiles are shown in Figure S1. Complement pathway biomarker data, as measured by the Wieslab, factor Bb and sC5b‐9 levels, indicated that full target engagement of factor B by iptacopan was sustained throughout the Part A treatment period for most patients in both the ITP (Figure S2A–C) and CAD cohorts (Figure S3A–C), whereas no significant impact on complement components C3 (Figures S2D,S3D) and C4d was observed (Figures S2E,S3E). A correlative analysis of baseline clinical and laboratory variables with different efficacy endpoints to identify potential predictive biomarkers did not reveal any consistent trends (data not shown).

### 3.5 FACIT‐Fatigue

The median FACIT‐Fatigue scale remained largely unchanged in the ITP cohort, with a mean score at baseline of 34.4 (range: 12–52; *n* = 8), a mean score at week 12 of 22.7 (range: 9–33; *n* = 3), and a mean change from baseline of −3.0 (range: −10 to 4; *n* = 3) (Figure S4A). Conversely, in the CAD cohort, treatment with iptacopan resulted in a clinically meaningful improvement in the mean FACIT‐Fatigue score, with a mean score at baseline of 34.3 (range: 16–44; *n* = 10), a mean score at week 12 of 42.0 (range: 27–49; *n* = 9), and a mean increase from baseline of 8.8 (range: 2.0–16.0) (Figure S4B). These changes were maintained in most patients in Part B.

### 3.6 Safety

Treatment‐emergent adverse events (TEAEs) are summarized in Table 3. Seven patients (77.8%) in the ITP cohort and nine (90%) in the CAD cohort experienced at least one TEAE, most of which were of mild severity. The most frequently reported TEAEs (> 15% across all patients, irrespective of relatedness to study drug) were headache, asthenia (only reported in patients with CAD), nausea, and petechiae (only reported in patients with ITP). No deaths, infections from encapsulated bacteria, or drug‐related serious adverse events (SAEs) were reported. One severe drug‐related AE (eczema) in an ITP patient led to dose interruption. One CAD patient with a prior history of chronic renal insufficiency experienced two treatment‐emergent, non‐study drug‐related SAEs of blood creatinine increased and acute kidney injury. The same patient subsequently experienced study drug‐related TEAEs of alanine aminotransferase and aspartate aminotransferase increased which led to treatment discontinuation.

**TABLE 3:** Overview of AEs and TEAEs by preferred term (in ≥ 2 patients considering both cohorts)‐Part A and B.

Category	ITP cohort	CAD cohort	All patients
	*N* = 9	*N* = 10	*N* = 19
	*n* (%)	*n* (%)	*n* (%)
Adverse events	7 (77.8)	9 (90.0)	16 (84.2)
Treatment‐related	2 (22.2)	3 (30.0)	5 (26.3)
Severe AEs	1 (11.1)	0	1 (5.3)
Treatment‐related	1 (11.1)	0	1 (5.3)
SAEs	0	1 (10.0)	1 (5.3)
Treatment‐related	0	0	0
Fatal SAEs	0	0	0
Treatment‐related	0	0	0
AEs leading to discontinuation	0	2 (20.0)	2 (10.5)
Treatment‐related	0	1 (10.0)	1 (5.3)
AEs leading to dose adjustment/interruption	1 (11.1)	0	1 (5.3)
AEs requiring additional therapy	5 (55.6)	7 (70.0)	12 (63.2)
TEAEs (in ≥ 2 patients considering both cohorts)			
Headache	1 (11.1)	3 (30.0)	4 (21.1)
Asthenia	0	3 (30.0)	3 (15.8)
Nausea	1 (11.1)	2 (20.0)	3 (15.8)
Petechiae	3 (33.3)	0	3 (15.8)
Back pain	0	2 (20.0)	2 (10.5)
COVID‐19	1 (11.1)	1 (10.0)	2 (10.5)
Constipation	0	2 (20.0)	2 (10.5)
Cough	0	2 (20.0)	2 (10.5)
Depressed mood	0	2 (20.0)	2 (10.5)
Hypokalemia	1 (11.1)	1 (10.0)	2 (10.5)
Nasopharyngitis	0	2 (20.0)	2 (10.5)
Pyrexia	0	2 (20.0)	2 (10.5)
Upper respiratory tract infection	1 (11.1)	1 (10.0)	2 (10.5)

*Note:* Numbers (*n*) represent counts of subjects; AEs occurring during treatment or within 7 days of the last study medication are summarized.

Abbreviations: AE, adverse events; CAD, cold agglutinin disease; ITP, immune thrombocytopenia; SAEs, serious adverse events; TEAEs, treatment emergent adverse events.

## 4 Discussion

This phase 2 basket trial was the first study to evaluate an oral complement inhibitor in patients with either ITP or CAD, and the first to investigate selective inhibition of the alternative complement pathway as a therapeutic strategy in these disorders. We found that the prespecified criteria for positive efficacy were met in the CAD cohort, but not in the ITP cohort. The positive results in CAD are consistent with complement serving as the central pathophysiologic mechanism of red cell destruction in CAD and provide clinical proof‐of‐concept for targeting the alternative complement pathway (and amplification loop) in an antibody‐mediated autoimmune disease. Conversely, the observed lack of efficacy in ITP underscores the importance of considering disease‐specific biology for targeted therapeutic intervention. Although a phase 1 study with sutimlimab suggested that inhibition of the classical complement pathway can meaningfully increase the platelet count in patients with chronic/refractory ITP [8], our data indicate that targeting the alternative complement pathway is less effective, suggesting that this pathway plays a lesser role in the pathophysiology of ITP.

Most patients in the CAD cohort achieved improvements in hemoglobin with iptacopan treatment, and half achieved the protocol‐defined primary endpoint. Hemoglobin responses were prompt and sustained and accompanied by normalization of LDH levels, suggesting good control of intravascular hemolysis. Improvements were also observed in other hemolytic parameters, including bilirubin (suggesting reduced extravascular hemolysis), haptoglobin and reticulocyte counts. Importantly, iptacopan treatment also brought about a clinically meaningful improvement in quality of life as assessed by the FACIT‐Fatigue scale that persisted throughout Part A and Part B of the study, exceeding the minimal clinically important difference of ≥ 5 points in the FACIT‐Fatigue scale for patients with CAD [30, 31, 32].

Though the comparability is limited by different study designs, these results are largely consistent with the clinical data observed with other complement inhibitors in CAD, including the intravenously administered monoclonal antibody sutimlimab [13, 14] and the complement C3 inhibitor pegcetacoplan [15], administered via subcutaneous infusion pump. As compared to the clinical data observed in PNH, where treatment with iptacopan effectively controls both intra‐ and extravascular hemolysis [33], there appears to be some degree of persistent hyperbilirubinemia, despite excellent control of LDH levels. This is likely mediated by the unchanged C3 fragment deposition observed on RBCs and suggests that, in CAD, where complement activation is initiated via the classical pathway, iptacopan (an inhibitor of the alternative complement pathway and amplification loop) acts primarily by preventing C5 convertase formation and intravascular (MAC‐mediated) hemolysis, with a potentially lesser impact on C3 convertase formation and extravascular (opsonin‐mediated) hemolysis.

Finally, the PK profile and exposure parameters of iptacopan were broadly comparable across ITP and CAD cohorts and also similar to those observed in healthy subjects and other patient populations [33]. In general, inter‐patient variability in exposure to iptacopan was low to moderate with %CV between 15% and 35%. Complement pathway biomarker data indicated full target engagement was sustained throughout the Part A treatment period for most patients in both cohorts.

Overall, iptacopan demonstrated a favorable safety profile in both ITP and CAD cohorts, in line with the observations in other clinical studies with iptacopan [23, 25, 34, 35, 36]. No infections attributed to encapsulated bacteria, particularly no meningococcal or pneumococcal infections, were reported. This is consistent with previous reports, suggesting that alternative complement pathway inhibition does not increase susceptibility to meningococcal and pneumococcal infection in vaccinated individuals [37, 38]. Overall, the data in this study did not reveal any new safety risks.

In conclusion, in this phase 2 basket trial, iptacopan monotherapy showed encouraging efficacy in patients with CAD, with clinically meaningful improvements in hemoglobin, markers of hemolysis, and health‐related quality of life as measured by FACIT‐Fatigue. Iptacopan was not efficacious in ITP, possibly reflective of the more limited role of complement in autoimmune platelet destruction and the complex pathophysiology of ITP. Overall, iptacopan was generally well tolerated, and no new safety signals were identified in this study. The results seen in patients with CAD are encouraging but require confirmation in a larger study.

## Author Contributions

**Alexander Röth:** acquisition of data, analysis and interpretation of data, provision of study material or patients, manuscript writing, manuscript reviewing, and manuscript approval. **Wilma Barcellini:** acquisition of data, analysis and interpretation of data, provision of study material or patients, manuscript writing, manuscript reviewing, and manuscript approval. **Christine Ademokun:** acquisition of data, analysis and interpretation of data, provision of study material or patients, manuscript writing, manuscript reviewing, and manuscript approval. **Junho Jang:** acquisition of data, analysis and interpretation of data, provision of study material or patients, manuscript writing, manuscript reviewing, and manuscript approval. **Maria Luisa Lozano:** acquisition of data, analysis and interpretation of data, provision of study material or patients, manuscript writing, manuscript reviewing, and manuscript approval. **David Valcarcel Ferreiras:** acquisition of data, analysis and interpretation of data, provision of study material or patients, manuscript writing, manuscript reviewing, and manuscript approval. **Cristina Pascual‐Izquierdo:** acquisition of data, analysis and interpretation of data, provision of study material or patients, manuscript writing, manuscript reviewing, and manuscript approval. **Shripad Chitnis:** conception and design, acquisition of data, analysis and interpretation of data, manuscript writing, manuscript reviewing, and manuscript approval. **Sofiya Matviykiv:** conception and design, acquisition of data, analysis and interpretation of data, manuscript writing, manuscript reviewing, and manuscript approval. **Alessandra Vitaliti:** conception and design, acquisition of data, analysis and interpretation of data, manuscript writing, manuscript reviewing, and manuscript approval. **Chi Chen:** conception and design, acquisition of data, analysis and interpretation of data, manuscript writing, manuscript reviewing, and manuscript approval. **Vasiliki Katsanou:** conception and design, acquisition of data, analysis and interpretation of data, manuscript writing, manuscript reviewing, and manuscript approval. **Raghav Chawla:** conception and design, acquisition of data, analysis and interpretation of data, manuscript writing, manuscript reviewing, and manuscript approval. **Hanny Al‐Samkari:** conception and design, acquisition of data, analysis and interpretation of data, provision of study material or patients, manuscript writing, manuscript reviewing, and manuscript approval.

## Funding

This work was supported by Novartis Pharmaceuticals.

## Ethics Statement

This study was conducted in accordance with the International Council for Harmonization of Technical Requirements for Registration of Pharmaceuticals for Human Use (ICH) Harmonized Tripartite Guidelines for Good Clinical Practice, with applicable local regulations, including European Directive 2001/20/EC and US Code of Federal Regulations Title 21, and the Declaration of Helsinki. The protocol and all amendments were reviewed and approved by a properly constituted institutional review board/independent ethics committee/research ethics board (IRB/IEC/REB) before study start. Eligible patients provided written, IRB/IEC/REB‐approved informed consent prior to study start.

## Consent

Eligible patients provided written, IRB/IEC/REB‐approved informed consent prior to study start.

## Conflicts of Interest

Alexander Röth: Consultant to Alexion Pharmaceuticals, Amgen, Apellis, Bioverativ, BioCryst, F. Hoffmann‐La Roche Ltd., Kira, Novartis, Sanofi, and Sobi; received research funding from Roche; and received honoraria from Alexion, F. Hoffmann‐La Roche Ltd., Grifols, Sanofi, and Sobi. Wilma Barcellini: Consultancy/Advisory Board (Agios, Alexion, Apellis, Incyte, Momenta, Novartis, Roche, Sanofi, SOBI); lecture fee/congress support (Alexion, Incyte, Novartis, Sanofi); research support (Alexion). Maria Luisa Lozano: Consultancy fees from Amgen, Novartis, Grifols, Argenx, Sanofi and Sobi. David Valcarcel Ferreiras: Honoraria/Expenses from Abbvie, Agios, Amgen, Asofarma, Astellas, Celgene/BMS, Grifols, Jansen, Jazz, MSD, Novartis, Pfizer, Sanofi, Sobi and Takeda. On Consulting/Advisory Board of Abbvie, Amgen, Astellas, Celgene/BMS, Jazz, Novartis, Sanofi, Servier, Sobi, Syros, Takeda. Funded Research from Agios and Celgene/BMS. Meetings, travel, accommodation from Abbvie, Agios, Amgen, Celgene/BMS, Grifols and Jazz. Cristina Pascual‐Izquierdo: Consulting fees from Amgen, Novartis, Sanofi, Sobi, Werfen and Takeda. Shripad Chitnis is a Novartis employee. Sofiya Matviykiv is a Novartis employee. Alessandra Vitaliti is a Novartis employee. Chi Chen is a Novartis employee. Vasiliki Katsanou is a Novartis employee. Raghav Chawla is a Novartis employee. Hanny Al‐Samkari: Research funding to institution (Agios, Amgen, Novartis, Sobi, Vaderis); consultancy (Agios, Alnylam, Amgen, Alpine, Argenx, Novartis, Pharmacosmos, Sanofi, Sobi). The other authors declare no conflicts of interest.

## Supporting information


**Data S1:** Supporting Information.

## Data Availability

Novartis is committed to sharing access to patient‐level data and supporting clinical documents from eligible studies with qualified external researchers. These requests are reviewed and approved by an independent review panel based on scientific merit. All data provided are anonymized to respect the privacy of patients who have participated in the trial in line with applicable laws and regulations. This trial data availability is according to the criteria and process described on www.clinicalstudydatarequest.com.
