# Supplementary material for: Iptacopan for Immune Thrombocytopenia and Cold Agglutinin Disease: A Global Phase 2 Basket Clinical Trial
Source: Am J Hematol. 2025 Dec 9;101(2):242–54. doi: 10.1002/ajh.70147 (PMC12766356; doi:10.1002/ajh.70147)
Supplement: Supplementary file 1 — Data S1: Supporting Information. [file AJH-101-242-s001.docx]

**Supplementary materials for manuscript titled “Iptacopan for immune thrombocytopenia and cold agglutinin disease: a global phase 2 basket clinical trial”**

**TABLE OF CONTENTS**

| **SECTION** | **PAGE NUMBER** |
| --- | --- |
| Supplementary Methods | 2-3 |
| Complement Pathway Biomarker Assay  sC5b-9  Factor Bb  Wieslab  C4d | 2 |
| C3 and C4  C3 methodology  C4 methodology | 2-3 |
| Disease biomarkers methodology  LDH  Total Bilirubin  Haptoglobin  Reticulocytes  C3d deposition on RBC | 3 |
| Supplementary Figures and Tables  Figure S1: Individual and arithmetic mean plasma concentration-time profiles- Part A (PK analysis set)  Figure S2-: Complement pathway biomarkers in ITP cohort- Part A.  Figure S3- Complement pathway biomarkers in CAD cohort- Part A  Figure S4- Individual patient and arithmetic mean data FACIT-Fatigue score over time  Table S1- Vaccinations against encapsulated bacteria - Part A & B  Table S2: Number of prior RBC transfusions at baseline.  Table S3- Demographic and other baseline characteristics (Part B)  Table S4- Patient disposition - Part A & B  Table S5- Summary of PK parameters for both the cohorts | 4-11  4  5  6  7  8  8-9  9-10  10  11 |

**SUPPLEMENTARY MATERIALS**

**SUPPLEMENTARY METHODS:**

**Complement pathway biomarker assay**

**sC5b-9:** Quantitative determination of soluble C5b-9 (sC5b-9) in human K2-EDTA plasma was done by ELISA using a commercial kit (MicroVueTM SC5b-9 Plus Enzyme ImmunoAssay kit, Ref. No. A029, Quidel® Corporation, distributed by Eurobio Scientific and by QuidelOrtho from June 2023). The developed method is based on a sandwich ELISA using microtiter plate pre-coated with a mouse monoclonal antibody that specifically binds to the C9 ring of sC5b-9. After calibration standards (STDs), quality controls (QCs) and samples incubation, a wash cycle removes any unbound material. Bound sC5b-9 is subsequently detected with a goat polyclonal antibody conjugated to horseradish peroxidase, which binds to antigens of the sC5b-9 complex. Following a wash to remove any unbound antibody, a mix solution of QuantaBluTM substrate solution and QuantaBluTM stable peroxide solution is added. The peroxidase activity is stopped by adding QuantaBluTM stop solution and the relative fluorescence units (RFU) are measured. The excitation and emission wavelengths for QuantaBluTM substrate are 325 nm and 420 nm, respectively. Samples are measured in duplicate and results are expressed in ng/mL.

**Factor Bb:** Quantitative determination of circulating Bb fragment of factor B by ELISA was done using a commercial kit (MicroVueTM complement Bb Plus Fragment EIA, Ref. No. A027, Quidel® Corporation, distributed by Eurobio Scientific and by QuidelOrtho from June 2023). The method is based on a sandwich ELISA. A mouse monoclonal antibody against human Bb is used as capture reagent. Bb is detected using horseradish peroxidase (HRP) conjugated murine anti-Bb antibody and a chromogenic substrate. Samples are measured in duplicate, and results are expressed in ng/mL.

**Wieslab:** Measurement of Wieslab complement activity (alternative pathway) was done using a commercial kit (Wieslab® complement system (alternative pathway), Ref. No. COMPL AP 330, Svar Life Science). Wieslab® complement AP assay combines principles of the haemolytic assay for complement activation with the use of labelled antibodies specific for the neoantigen produced (C5b-9) as a result of complement activation. The amount of C5b-9 generated is proportional to the functional activity of complement pathways. Samples are measured in duplicate, and results are expressed in percentage.

**C4d:** Quantitative determination of C4d by immunoassay was done using a commercial kit (Ref. No. MSPPCOMPLC4DRUO; Svar Lifesciences). The method is based on a colorimetric sandwich ELISA. Samples are diluted in assay diluent with 0.05% Tween-20 and transferred to the microtiter wells. During this first incubation C4d in the sample is captured by the anti-C4d-Neo monoclonal antibody, pre-coated on the surface of the microtiter wells. After washing to remove unbound material, a second horseradish peroxidase (HRP) labelled monoclonal antibody, that binds to both allelic variants of C4d (A and B), is added the to the well. After incubation the wells are washed again, and a substrate is added and incubated. The color development is stopped after 45 minutes, and the color is measured in a spectrophotometer. The color is directly proportional to the amount of C4d bound to the well. The amount of C4d is determined by comparison to the color development of the calibrator samples. Samples were measured in duplicate, and results were expressed in ng/mL.

**C3 and C4 were measured in serum samples**

**C3 methodology:** The Diagnostic Kit “Architect/Aeroset Complement C3” from Abbott Diagnostics was used with the Abbott Architect ci16200 instrument. The C3 assay is an immunoturbidimetric procedure that measures increasing sample turbidity caused by the formation of insoluble immune complexes when antibody to C3 is added to the sample. Sample containing C3 is incubated with a buffer and a sample blank determination is performed prior to the addition of C3 antibody. In the presence of an appropriate antibody in excess, the C3 concentration is measured as a function of turbidity.

**C4 methodology:** The diagnostic kit “Architect/Aeroset Complement C4” from Abbott Diagnostics was used with the Abbott Architect system. The C4 assay is an immunoturbidimetric procedure that measures increasing sample turbidity caused by the formation of insoluble immune complexes when antibody to C4 is added to the sample. Sample containing C4 is incubated with a buffer (R1) and a sample blank determination is performed prior to the addition of C4 antibody R2). In the presence of an appropriate antibody in excess, the C4 concentration is measured as a function of turbidity.

**Disease biomarkers methodology**

**LDH:** LDH was measured in serum using the diagnostic kit: “Architect Lactate Dehydrogenase” from Abbott Diagnostics and with the Abbott ARCHITECT System. This method uses the IFCC Recommended, forward reaction - Lactate to Pyruvate. Lactate dehydrogenase is a hydrogen transfer enzyme that catalyzes the oxidation of L-lactate to pyruvate with the mediation of NAD+ as a hydrogen acceptor. L-Lactate + NAD+ f*orward Reaction of Lactate Dehydrogenase* to Pyruvate + NADH H+

**Total Bilirubin:** Total bilirubin was measured in serum using the diagnostic kit: “Architect/Aeroset Total Bilirubin” from Abbott Diagnostics with the Abbott ARCHITECT System. The methodology used was Diazonium Salt - Traditional methods of measuring bilirubin are based on the reaction of bilirubin with a diazo reagent to form the colored compound azobilirubin. The diazo reaction can be accelerated by the addition of various chemicals. For example, Malloy-Evelyn used methanol, Jendrassik-Gróf used caffeine, and Walters-Gerarde used dimethyl sulfoxide (DMSO). Modifications of these methods included the addition of surfactants as solubilizing agents.

Total (conjugated and unconjugated) bilirubin couples with a diazo reagent in the presence of a surfactant to form azobilirubin. The diazo reaction is accelerated by the addition of surfactant as a solubilizing agent. The increase in absorbance at 548 nm due to azobilirubin is directly proportional to the total bilirubin concentration.

**Haptoglobin:** Haptoglobin was measured in serum using the diagnostic kit: “Architect Haptoglobin” from Abbott Diagnostics with Abbott ARCHITECT System. The Haptoglobin assay is an immunoturbidimetric procedure that measures increasing sample turbidity caused by the formation of insoluble immune complexes when antibody to haptoglobin is added to the sample. Sample containing haptoglobin is incubated with a buffer (Reagent 1) and a sample blank determination is performed prior to the addition of haptoglobin antibody (Reagent 2). In the presence of an appropriate antibody in excess, the haptoglobin concentration is measured as a function of turbidity.

**Reticulocytes:** Reticulocytes were measured in entire blood samples using the diagnostic kit: “Reticulocyte Reagent” from Siemens Healthcare Diagnostics Limited with Siemens Healthcare Diagnostics ADVIA 2120Instrument. Two microliters of and EDTA anticoagulated whole-blood sample are mixed online with the ADVIA 2120 autoRETIC reagent.  The ADVIA 2120 autoRETIC reagent (oxazine 750) isovolumetrically spheres the erythroid cells and stains cellular RNA.  Low-angle laser light scatter, high-angle laser light scatter, and absorption characteristics of all cells are counted and measured.  The absorption data are used to classify each cell as a reticulocyte or mature red blood cell based on its RNA content.

**C3d deposition on RBC:** C3d deposition on RBC was assessed by flow cytometry using whole blood with no prior lysis or wash steps. The assay quantified the percentage of CD235a+ C3d+ RBC. Whole blood samples were stained with fluorescently labeled antibodies and acquired on a FACSCanto™ II flow cytometer using FACSDiva™. Data were analyzed with FCS Express™ (De Novo Software).

**Supplementary figures and tables**

***Figure S1:* Individual and arithmetic mean plasma concentration-time profiles - Part A (PK analysis set**). (A) ITP cohort, Day 15 and Day 57. (B) CAD cohort, Day 15 and Day 57. CAD, cold agglutinin disease; ITP, immune thrombocytopenia.

***Figure S2:* Complement pathway biomarkers over time in patients with ITP - Part A (A) Wieslab. (B) factor Bb (C) sC5b-9 Levels. (D) Complement C3. (E) C4d.** Values below the LLOQ are imputed as LLOQ/2, values above the ULOQ are imputed as ULOQ. Actual study days are shown for individual patients, whereas planned study visit days were considered for the calculation of mean and confidence interval.

*Visits occurring outside of the protocol-defined visit schedule and early end-of-treatment visits have been matched to and are displayed as their closest planned visit as per the time window defined in the SAP. The confidence intervals for the raw value of biomarkers (LLN/LLOQ of which is non-negative) are adjusted such that the negative lower limit is truncated to 0.

ITP, immune thrombocytopenia; LLN/LLOQ, lower limit of normal/lower limit of quantification; ULOQ, upper limit of normal; SAP, statistical analysis plan.

***Figure S3:* Complement pathway biomarkers over time in patients with CAD - Part A. A) Wieslab. (B) factor Bb (C) sC5b-9 Levels. (D) Complement C3. (E) C4d.** Values below the LLOQ are imputed as LLOQ/2, values above the ULOQ are imputed as ULOQ. Actual study days are shown for individual patients, whereas planned study visit days were considered for the calculation of mean and confidence interval.

*Visits occurring outside of the protocol-defined visit schedule and early end-of-treatment visits have been matched to and are displayed as their closest planned visit as per the time window defined in the SAP. The confidence intervals for the raw value of biomarkers (LLN/LLOQ of which is non-negative) are adjusted such that the negative lower limit is truncated to 0. CAD, cold agglutinin disease; LLN/LLOQ, lower limit of normal/lower limit of quantification; ULOQ, upper limit of normal; SAP, statistical analysis plan

***Figure S4:* Individual patient and arithmetic mean data FACIT-Fatigue score over time. (A) ITP COHORT. (B) CAD COHORT.** *Visits occurring outside of the protocol-defined visit schedule and early end-of-treatment visits have been matched to and are displayed as their closest planned visit as per the time window defined in the SAP. CAD, cold agglutinin disease; ITP, immune thrombocytopenia; SAP, statistical analysis plan.

***Table S1:* Vaccinations against encapsulated bacteria - part A & B**

|  | **Cohort 1 (ITP)** | **Cohort 2 (CAD)** | **All patients** |
| --- | --- | --- | --- |
|  | **N=9** | **N=10** | **N=19** |
|  | **n (%)** | **n (%)** | **n (%)** |
| Number of subjects with at least one medication and significant non-drug therapies | 9 (100) | 10 (100) | 19 (100) |
| **Meningococcal vaccines** | 9 (100) | 10 (100) | 19 (100) |
| Meningococcal vaccine A/C/Y/W Conj (Tet Tox) | 3 (33ꞏ3) | 8 (80ꞏ0) | 11 (57ꞏ9) |
| Meningococcal vaccine B Rfhbp/Nada/Nhba Omv | 4 (44ꞏ4) | 3 (30ꞏ0) | 7 (36ꞏ8) |
| Meningococcal vaccine | 4 (44ꞏ4) | 2 (20ꞏ0) | 6 (31ꞏ6) |
| Meningococcal vaccine B Rfhbpa/Fhbpb | 0 | 4 (40ꞏ0) | 4 (21ꞏ1) |
| Meningococcal vaccine B | 1 (11ꞏ1) | 2 (20ꞏ0) | 3 (15ꞏ8) |
| Meningococcal vaccine A/C/Y/W | 2 (22ꞏ2) | 0 | 2 (10ꞏ5) |
| Meningococcal vaccine A/C/Y/W Polysacch | 1 (11ꞏ1) | 0 | 1 (5ꞏ3) |
| Meningococcal vaccine C | 1 (11ꞏ1) | 0 | 1 (5ꞏ3) |
| **Pneumococcal vaccines** | 9 (100) | 10 (100) | 19 (100) |
| Pneumococcal vaccine Polysacch 23v | 3 (33ꞏ3) | 6 (60ꞏ0) | 9 (47ꞏ4) |
| Pneumococcal vaccine | 5 (55ꞏ6) | 1 (10ꞏ0) | 6 (31ꞏ6) |
| Pneumococcal vaccine Conj 13v (Crm197) | 2 (22ꞏ2) | 3 (30ꞏ0) | 5 (26ꞏ3) |
| Pneumococcal vaccine 13v | 1 (11ꞏ1) | 0 | 1 (5ꞏ3) |
| Pneumococcal vaccine Conj 7v (Crm197) | 0 | 1 (10ꞏ0) | 1 (5ꞏ3) |
| ***Haemophilus influenzae* B vaccines** | 7 (77ꞏ8) | 8 (80ꞏ0) | 15 (78ꞏ9) |
| Hib vaccine conj (Tet Tox) | 4 (44ꞏ4) | 6 (60ꞏ0) | 10 (52ꞏ6) |
| Hib vaccine | 2 (22ꞏ2) | 1 (10ꞏ0) | 3 (15ꞏ8) |
| *Haemophilus influenzae* B vaccines | 0 | 1 (10ꞏ0) | 1 (5ꞏ3) |
| Hib vaccine conj | 1 (11ꞏ1) | 0 | 1 (5ꞏ3) |

A medication can appear with more than one ATC class.
WHODD version: 202209.

***Table S2:*** **Number of prior RBC transfusions at baseline (CAD cohort)**

| **Patient** | **Number of RBC transfusions within 6 months before the start of iptacopan** | **Number of RBC transfusions within 12 months before the start of iptacopan** |
| --- | --- | --- |
| Patient 1 | 0 | 0 |
| Patient 2 | 1 | 1 |
| Patient 3 | 0 | 0 |
| Patient 4 | 0 | 0 |
| Patient 5 | 0 | 1 |
| Patient 6 | 0 | 0 |
| Patient 7 | 0 | 1 |
| Patient 8 | 14 | 27 |
| Patient 9 | 0 | 0 |
| Patient 10 | 0 | 2 |
| Mean (across all patients) | 1.5 | 3.2 |
| Median (range) (across all patients) | 0 (0-14) | 0.5 (0-27) |

CAD, cold agglutinin disease; RBC, red blood cell.

***Table S3:* Demographic and other baseline characteristics (part B)**

| **Characteristic** | **Cohort 1 (ITP) - SC5B-9 high** | **Cohort 1 (ITP) - all** | **Cohort 2 (CAD)** | **All patients** |
| --- | --- | --- | --- | --- |
| **Categories/statistics** | **N=1** | **N=1** | **N=8** | **N=9** |
| Age (years) |  |  |  |  |
| Mean (SD) | 42ꞏ0 | 42ꞏ0 | 64ꞏ3 (9ꞏ27) | 61ꞏ8 (11ꞏ41) |
| Median | 42ꞏ0 | 42ꞏ0 | 62ꞏ5 | 61ꞏ0 |
| Min-max | 42–42 | 42–42 | 52–77 | 42–77 |
|  |  |  |  |  |
| Sex - n (%) |  |  |  |  |
| Female | 0 | 0 | 8 (100) | 8 (88ꞏ9) |
| Male | 1 (100) | 1 (100) | 0 | 1 (11ꞏ1) |
|  |  |  |  |  |
| Race - n (%) |  |  |  |  |
| White | 1 (100) | 1 (100) | 8 (100) | 9 (100) |
|  |  |  |  |  |
|  |  |  |  |  |
| Number of prior therapies - n |  |  |  |  |
| n | 1 | 1 | 8 | 9 |
| Mean (SD) | 3ꞏ0 | 3ꞏ0 | 2ꞏ5 (1ꞏ60) | 2ꞏ6 (1ꞏ51) |
| Median | 3ꞏ0 | 3ꞏ0 | 2ꞏ0 | 2ꞏ0 |
| Min-max | 3–3 | 3–3 | 1–6 | 1–6 |
| Lactate dehydrogenase (U/L) |  |  |  |  |
| n | NA | NA | 6 | 6 |
| Mean (SD) |  |  | 452ꞏ8 (141ꞏ52) | 452ꞏ8 (141ꞏ52) |
| Median |  |  | 469ꞏ0 | 469ꞏ0 |
| Min-max |  |  | 279–598 | 279–598 |
|  |  |  |  |  |
| Platelets (10^9^/L) |  |  |  |  |
| n | 1 | 1 | NA | 1 |
| Mean (SD) | 18ꞏ0 | 18ꞏ0 |  | 18ꞏ0 |
| Median | 18ꞏ0 | 18ꞏ0 |  | 18ꞏ0 |
| Min-max | 18–18 | 18–18 |  | 18–18 |
|  |  |  |  |  |
| Haemoglobin (g/L) |  |  |  |  |
| n | NA | NA | 8 | 8 |
| Mean (SD) |  |  | 86ꞏ4 (10ꞏ23) | 86ꞏ4 (10ꞏ23) |
| Median |  |  | 87ꞏ0 | 87ꞏ0 |
| Min-max |  |  | 68–99 | 68–99 |
|  |  |  |  |  |
| Bilirubin (umol/L) |  |  |  |  |
| n | NA | NA | 8 | 8 |
| Mean (SD) |  |  | 54ꞏ5 (24ꞏ24) | 54ꞏ5 (24ꞏ24) |
| Median |  |  | 48ꞏ1 | 48ꞏ1 |
| Min-max |  |  | 30–98 | 30–98 |
|  |  |  |  |  |
| sC5b-9 (ug/L) ^ |  |  |  |  |
| N | 1 | 1 | NA | 1 |
| Mean (SD) | 312ꞏ9 | 312ꞏ9 |  | 312ꞏ9 |
| Median | 312ꞏ9 | 312ꞏ9 |  | 312ꞏ9 |
| Min-max | 313–313 | 313–313 |  | 313–313 |
|  |  |  |  |  |
| Number of background therapies - n |  |  |  |  |
| n | 1 | 1 | NA | 1 |
| Mean (SD) | 1ꞏ0 | 1ꞏ0 |  | 1ꞏ0 |
| Median | 1ꞏ0 | 1ꞏ0 |  | 1ꞏ0 |
| Min-max | 1–1 | 1–1 |  | 1–1 |

^Data for sC5b-9 at screening are summarised. SD, standard deviation. CAD, cold agglutinin disease; ITP, immune thrombocytopenia; SD, standard deviation.

***Table S4:* Patient disposition - part A & B**

|  | **Cohort 1 (ITP) - SC5B-9 high** | | **Cohort 1 (ITP) - SC5B-9 low** | | **Cohort 1 (ITP) - all** | | **Cohort 2 (CAD)** | | **All patients** | |
| --- | --- | --- | --- | --- | --- | --- | --- | --- | --- | --- |
|  | **N=4** | | **N=5** | | **N=9** | | **N=10** | | **N=19** | |
| **Disposition/reason** | **n (%)** | | **n (%)** | | **n (%)** | | **n (%)** | | **n (%)** | |
| **Part A** |  | |  | |  | |  | |  | |
| Subjects treated | 4 (100) | | 5 (100) | | 9 (100) | | 10 (100) | | 19 (100) | |
| Completed treatment | 2 (50ꞏ0) | | 2 (40ꞏ0) | | 4 (44ꞏ4) | | 9 (90ꞏ0) | | 13 (68ꞏ4) | |
| Completed follow-up and entered treatment part B | 1 (25ꞏ0) | | 0 | | 1 (11ꞏ1) | | 8 (80ꞏ0) | | 9 (47ꞏ4) | |
| Completed follow-up and did not enter treatment part B | 1 (25ꞏ0) | | 2 (40ꞏ0) | | 3 (33ꞏ3) | | 1 (10ꞏ0) | | 4 (21ꞏ1) | |
| Discontinued from treatment or study | 2 (50ꞏ0) | | 3 (60ꞏ0) | | 5 (55ꞏ6) | | 1 (10ꞏ0) | | 6 (31ꞏ6) | |
| Discontinued from treatment and had follow-up | 1 (25ꞏ0) | | 3 (60ꞏ0) | | 4 (44ꞏ4) | | 1 (10ꞏ0) | | 5 (26ꞏ3) | |
| Discontinued from treatment and did not have follow-up | 1 (25ꞏ0) | | 0 | | 1 (11ꞏ1) | | 0 | | 1 (5ꞏ3) | |
| Reason for discontinuation |  | |  | |  | |  | |  | |
| Adverse event | | 0 | 0 | | 0 | | 1 (10ꞏ0) | | 1 (5ꞏ3) | |
| Lack of efficacy | | 0 | 3 (60ꞏ0) | | 3 (33ꞏ3) | | 0 | | 3 (15ꞏ8) | |
| Protocol deviation | | 1 (25ꞏ0) | 0 | | 1 (11ꞏ1) | | 0 | | 1 (5ꞏ3) | |
| Subject decision | | 1 (25ꞏ0) | 0 | | 1 (11ꞏ1) | | 0 | | 1 (5ꞏ3) | |
|  |  | |  | |  | |  | |  | |
| **Part B** |  | |  | |  | |  | |  | |
| Subjects treated | 1 (25ꞏ0) | | 0 | | 1 (11ꞏ1) | | 8 (80ꞏ0) | | 9 (47ꞏ4) | |
| Discontinued from treatment or study | 1 (25ꞏ0) | | 0 | | 1 (11ꞏ1) | | 8 (80ꞏ0) | | 9 (47ꞏ4) | |
| Entered follow-up and discontinued from study | 1 (25ꞏ0) | | 0 | | 1 (11ꞏ1) | | 8 (80ꞏ0) | | 9 (47ꞏ4) | |
| Reason for discontinuation |  | |  | |  | |  | |  | |
| Adverse event | 0 | | 0 | | 0 | | 1 (10ꞏ0) | | 1 (5ꞏ3) | |
| Lack of efficacy | 0 | | 0 | | 0 | | 1 (10ꞏ0) | | 1 (5ꞏ3) | |
| Study terminated by sponsor | 1 (25ꞏ0) | | 0 | | 1 (11ꞏ1) | | 6 (60ꞏ0) | | 7 (36ꞏ8) | |

CAD, cold agglutinin disease; ITP, immune thrombocytopenia.

***Table S5:* Summary of PK parameters for both the cohorts**

| **Parameter** | **Statistics** | **ITP cohort N=9** | | **CAD cohort**  **(N=10)** | |
| --- | --- | --- | --- | --- | --- |
|  |  | **Day 15** | **Day 57** | **Day 15** | **Day 57** |
| AUC to last nonzero conc (AUClast; h*ng/mL) | n | 7 | 3 | 8 | 9 |
|  | Mean (SD) | 24200ꞏ0 (6110ꞏ00) | 21300ꞏ0 (5280ꞏ00) | 31100ꞏ0 (5830ꞏ00) | 28200ꞏ0 (6880ꞏ00) |
|  | CV% mean | 25ꞏ2 | 24ꞏ8 | 18ꞏ8 | 24ꞏ4 |
|  | Geo-mean | 23500 | 20800 | 30600 | 27500 |
|  | CV% geo-mean | 26ꞏ7 | 27ꞏ6 | 18ꞏ8 | 23ꞏ8 |
|  | Median | 23600 | 23800 | 31000 | 26700 |
|  | [Min; max] | [15100; 32900] | [15300; 24900] | [22600; 41800] | [20100; 40200] |
| AUC overdosing interval (AUC0-12h; h*ng/mL) | n | 7 | 3 | 8 | 9 |
|  | Mean (SD) | 24200ꞏ0 (6110ꞏ00) | 21300ꞏ0 (5280ꞏ00) | 31100ꞏ0 (5830ꞏ00) | 28200ꞏ0 (6880ꞏ00) |
|  | CV% mean | 25ꞏ2 | 24ꞏ8 | 18ꞏ8 | 24ꞏ4 |
|  | Geo-mean | 23500 | 20800 | 30600 | 27500 |
|  | CV% geo-mean | 26ꞏ7 | 27ꞏ6 | 18ꞏ8 | 23ꞏ8 |
|  | Median | 23600 | 23800 | 31000 | 26700 |
|  | [Min; max] | [15100; 32900] | [15300; 24900] | [22600; 41800] | [20100; 40200] |
| Max conc (Cmax; ng/mL) | n | 7 | 3 | 8 | 9 |
|  | Mean (SD) | 3190ꞏ0 (465ꞏ00) | 2940ꞏ0 (1020ꞏ00) | 4800ꞏ0 (838ꞏ00) | 4420ꞏ0 (1300ꞏ00) |
|  | CV% mean | 14ꞏ6 | 34ꞏ8 | 17ꞏ4 | 29ꞏ5 |
|  | Geo-mean | 3160 | 2810 | 4740 | 4260 |
|  | CV% geo-mean | 14ꞏ3 | 39ꞏ3 | 16ꞏ6 | 28ꞏ8 |
|  | Median | 3020 | 3090 | 4460 | 4170 |
|  | [Min; max] | [2700; 3910] | [1850; 3880] | [4010; 6420] | [2830; 7160] |
| Time of Cmax (Tmax; h) | n | 7 | 3 | 8 | 9 |
|  | Median | 2 | 2 | 1 | 1ꞏ97 |
|  | [Min; max] | [0ꞏ75; 5ꞏ15] | [2; 2] | [1; 2] | [1; 2] |

n=number of subjects with corresponding evaluable PK parameters.

AUC, area under curve; CAD, cold agglutinin disease; Cmax, maximum concentration of a drug in the bloodstream; CV%, coefficient of variation %; ITP, immune thrombocytopenia; PK, pharmacokinetics; SD, standard deviation; Tmax, time taken to reach maximum concentration of a drug in the bloodstream.
